# Supplementary material for: Chronic stimulation of group II metabotropic glutamate receptors in the medulla oblongata attenuates hypertension development in spontaneously hypertensive rats
Source: PLoS One. 2021 May 19;16(5):e0251495. doi: 10.1371/journal.pone.0251495 (PMC8133461; doi:10.1371/journal.pone.0251495)

This study provided the first evidence that mGluR2/3 as a particular crucial target in hypertensive development. Long-term compensating mGluR2/3 function chronically, particularly during the ages of hypertensive development, may attenuate the progression, which is mainly modulated by parasympathetic dominance which also brings better baroreflex function (Fig. 7). In cellular aspect, mGluR2 can be more directly associated with blood pressure regulation than mGluR3 (Fig.8). The finding supports the understanding of hypertensive development, which contributes the prevention and therapeutics on hypertension.


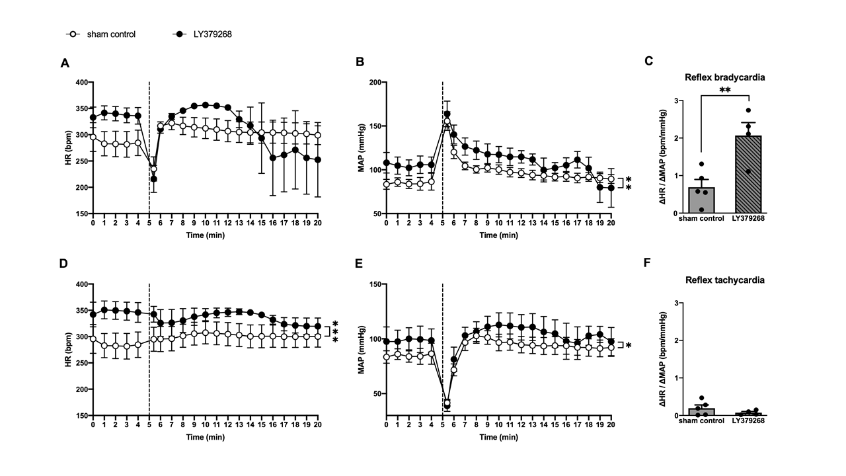

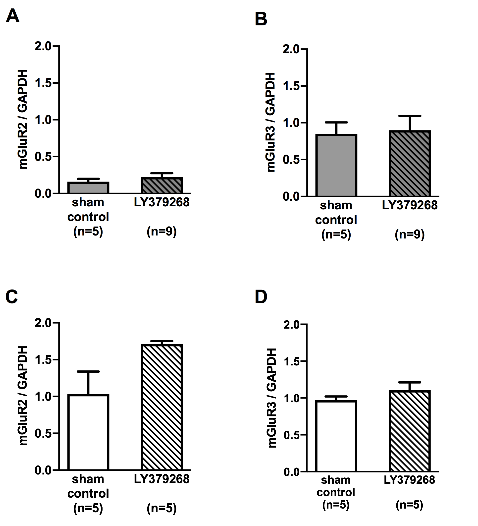


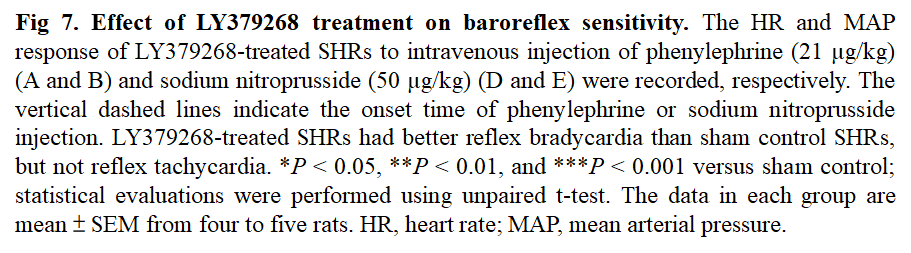

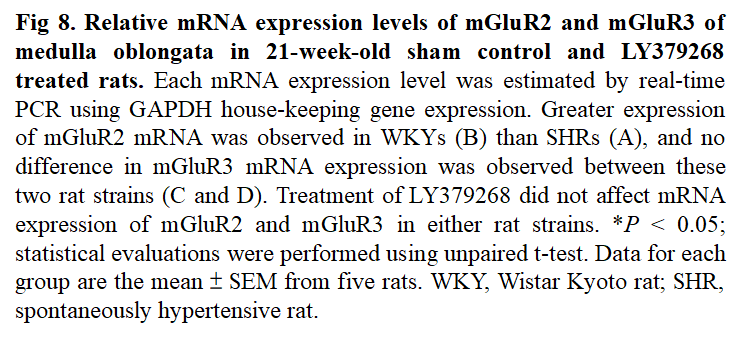

Supplement: S1 File — (DOCX) [file pone.0251495.s001.docx]
